# *Thauera aminoaromatica* MZ1T Identified as a Polyhydroxyalkanoate-Producing Bacterium within a Mixed Microbial Consortium

**DOI:** 10.3390/bioengineering7010019

**Published:** 2020-02-21

**Authors:** Dana I. Colpa, Wen Zhou, Jan Pier Wempe, Jelmer Tamis, Marc C. A. Stuart, Janneke Krooneman, Gert-Jan W. Euverink

**Affiliations:** 1 Products and Processes for Biotechnology Group, Engineering and Technology Institute Groningen, University of Groningen, Nijenborgh 4, 9747 AG Groningen, The Netherlands; 2 Paques Technology B.V., Tjalke de Boerstrjitte 24, 8561 EL Balk, The Netherlands; 3 Groningen Biomolecular Sciences and Biotechnology Institute, University of Groningen, Nijenborgh 7, 9747 AG Groningen, The Netherlands

**Keywords:** *Thauera aminoaromatica* MZ1T, biopolymers, polyhydroxyalkanoate (PHA), polyhydroxybutyrate (PHB), 16S-rDNA analysis, biodiversity, mixed microbial consortium

## Abstract

Polyhydroxyalkanoates (PHAs) form a highly promising class of bioplastics for the transition from fossil fuel-based plastics to bio-renewable and biodegradable plastics. Mixed microbial consortia (MMC) are known to be able to produce PHAs from organic waste streams. Knowledge of key-microbes and their characteristics in PHA-producing consortia is necessary for further process optimization and direction towards synthesis of specific types of PHAs. In this study, a PHA-producing mixed microbial consortium (MMC) from an industrial pilot plant was characterized and further enriched on acetate in a laboratory-scale selector with a working volume of 5 L. 16S-rDNA microbiological population analysis of both the industrial pilot plant and the 5 L selector revealed that the most dominant species within the population is *Thauera aminoaromatica* MZ1T, a Gram-negative beta-proteobacterium belonging to the order of the Rhodocyclales. The relative abundance of this *Thauera* species increased from 24 to 40% after two months of enrichment in the selector-system, indicating a competitive advantage, possibly due to the storage of a reserve material such as PHA. First experiments with *T. aminoaromatica* MZ1T showed multiple intracellular granules when grown in pure culture on a growth medium with a C:N ratio of 10:1 and acetate as a carbon source. Nuclear magnetic resonance (NMR) analyses upon extraction of PHA from the pure culture confirmed polyhydroxybutyrate production by *T. aminoaromatica* MZ1T.

## 1. Introduction

The production and use of fossil-based plastic materials became ubiquitous, especially after the second world war. Since then, plastics play an indispensable role in our daily life. However, in the past decades, it has become evident that these plastics persist in nature and have accumulated to incredibly high volumes and become severe ecological threats. The immense volume of the plastic soup in our oceanic ecosystems is one of the most eye-catching examples [1]. Currently, the global plastics production reached approximately 360 million tons and shows an increasing trend [1]. Obviously, the tide must be turned from an ecological point of view by (1) reducing the use of plastics, (2) recycling of existing plastics, (3) production of plastics that are not petrochemically derived and that are biodegradable once they end up in the environment. 

Polyhydroxyalkanoates (PHAs) are examples of bio-based polymers, produced from natural organic resources and organic waste streams by a variety of microorganisms [2]. PHAs are known to be biodegradable and biocompatible with many materials and tissues, thereby having potential in diverse applications. For example, PHAs have been used in drug delivery, in tissue engineering [3], and in agriculture in mulch films and nets [4].

The PHA-producing microbes utilize diverse organic carbon substrates for growth and to synthesize intracellular storage materials in the form of PHAs. The accumulation of PHA occurs mainly under high carbon-substrate levels combined with nutritionally imbalanced environmental conditions. PHA accumulation is a way to store energy and carbon in order to survive starvation-conditions in scarcer times [5]. Once the micro-organisms experience starvation, thus lacking sufficient external carbon source for growth, the accumulated PHA will be used as growth substrate [6,7]. A feast–famine regime is a proven approach to specifically enrich for PHA-producing organisms [8,9,10]. During the feast-phase (excess carbon), PHA accumulation occurs and during the famine-phase (no carbon) the PHA can be used as an alternative carbon source. Organisms that stored PHA have a competitive advantage over those that did not store reserve materials. 

Up until now, PHA-producing microorganisms were found in more than 70 archaeal and (cyano)bacterial genera [11,12]. Mixed microbial consortia are promising for accumulating PHAs because of their potential in using low-cost waste streams as substrates like wastewater, crude palm kernel oil, used cooking oil, cheese whey, and coffee grounds, as well as swine waste liquor [10,13,14,15,16,17]. After hydrolysis and acidogenic fermentation, most organic waste streams contain mixtures of (volatile) fatty acids, such as acetic acid, propionic acid, butyric acid, valeric acid, and hexanoic acid [18,19]. These fatty acids are perfect substrates for the production of PHAs, that consist of the polymerized hydroxylated fatty acid monomers. Mixtures of (volatile) fatty acids, as well as single fatty acids, could be used for PHA production. 

To date, there are over 150 different types of PHAs [11]. Most of the PHAs are classified based on their chain length and monomer composition [2,11]. Examples of short-chain length PHA (*scl*-PHA, 3–5 carbon atoms per monomer) are polyhydroxybutyrate (PHB), polyhydroxyvalerate (PHV), and the copolymer hydroxybutyrate-*co*-hydroxyvalerate (PHBV). Medium-chain length PHAs (*mcl*-PHAs) are characterized by a monomer composition based on larger 3-hydroxy fatty acids (6–14 carbon atoms per monomer) like polyhydroxyhexanoate and polyhydroxyoctanoate and copolymers thereof. As described above, when acetic acid is used as a sole carbon source, PHB accumulates inside the cells [20,21]. The presence and concentration of acetic acid in mixed substrates is, to some extent, thought to regulate the ratio of 3-hydroxybutyrate (3HB) in the produced copolymers [22]. This holds for the production of PHAs by pure or defined mixed cultures growing on defined (mixtures of) substrates. However, substrate-directed PHA production is not fully understood when using undefined microbial consortia and complex mixtures of substrates derived from organic waste streams [23]. So, one can imagine that for successful conversion of undefined low-cost organic waste into PHA using undefined natural microbial consortia, many challenges exist. For example, what is the effect of the feed-composition on the PHA production? What is the efficiency of the cells to produce PHA? Which yields can be achieved? Which types of PHAs can be produced, and how can this be controlled? Which microbes within the consortia are involved in the PHA production? Therefore, it is relevant to gain more insight into the mixed microbial consortia and to understand their dynamics in relation to changes in environmental conditions and substrate compositions. Furthermore, it is important to identify the predominant species involved in the PHA production and to understand their eco-physiological characteristics in general, and towards PHA production specifically. 

In this work, we aim to enrich, identify, isolate, and characterize the predominant bacterial species within a PHA-producing mixed microbial consortium. In our lab, we grew this microbial consortium to further enrich the predominant strain under a feast–famine regime. The substrate consumption and microbiological diversity was analyzed. Lastly, the predominant bacterial species was identified and characterized as a PHA-producing bacterial strain. 

## 2. Materials and Methods 

### 2.1. Strains and Chemicals

Media components, salts, and solvents were obtained from Sigma-(Aldrich) (St. Louis, MO, USA), Merck (Kenilworth, NJ, USA), Difco (Becton Dickinson Company, Franklin Lakes, NJ, USA), Biosolve Chimie (Dieuze, France), Becton Dickinson Company (Franklin Lakes, NJ, USA), and BOOM (Meppel, The Netherlands). The LCK 365 kit for organic acid detection was obtained from Hach Lange. The genomic DNA was extracted using the FastDNA Spin kit for soil from MP Biomedicals. *Thauera aminoaromatica* MZ1 (DSM No. 25461) was obtained from DSMZ (Braunschweig, Germany). 

### 2.2. Operating Conditions Pilot Reactor

**Figure 1:**
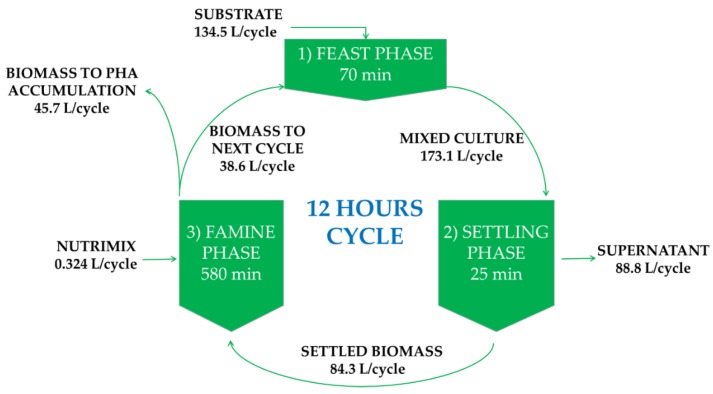
Operating conditions of the pilot selector reactor. The reactor was operated under a feast–famine regime with a cycle of 12 h. Leachate from organic waste was used as substrate.

The pilot reactor (working volume 180 L) was operated as an aerobic SBR (sequence batch reactor). Air was supplied via a fine bubble diffuser at a rate of 100 L/min to provide oxygen and ensure mixing. The dissolved oxygen concentration was kept above 2 mg/L. The reactor was kept at 30 ± 3 °C. The pH in the system was not controlled but remained between 8.0 and 8.5 due to the buffering capacity of the substrate. The substrate was leachate from organic waste with a soluble COD (chemical oxygen demand) content of 5 ± 0.6 g/L (average ± standard deviation, n = 62). The substrates’ COD content comprised 3 ± 1 g/L of volatile fatty acids: the fractions of individual n-VFA compared to the total n-VFA content were: acetic acid 25% ± 9%, propionic acid 15% ± 5%, butyric acid 29% ± 6% and valeric acid 32% ± 10% on COD basis. The remainder of the soluble COD (2 g/L) of COD could not be readily identified; nevertheless, it was observed that from the remaining fraction, about 1 ± 0.4 g/L was converted in the SBR cycle while the rest was inert. Total ammonium nitrogen concentrations in the substrate were around 300 mg/L. Additional nutrient solution (nutrimix) was added to prevent nitrogen and phosphorus limitation in the reactor. The substrate was fed pulse-wise in order to select for PHA-producing bacteria [24], with a cycle of 12 h, see Figure 1. Every cycle, the floc-forming PHA-producing bacteria were settled, and the upper half of the reactor volume (supernatant) was discarded. The remaining biomass was split in two: half of the volume was kept in the selector, and the other half was transferred to a fed-batch reactor for PHA accumulation. A part (5 L) of the broth of the pilot reactor was used to start the 5 L lab-scale bioreactor. 

### 2.3. Bacterial Growth Conditions—Lab-Scale Bioreactor

#### 2.3.1. Growth Media

The culture medium for the enrichment bioreactor consisted of 36 mM sodium acetate, 16.6 mM NH_4_Cl, 2.5 mM KH_2_PO_4_, 0.55 mM MgSO_4_, and 0.72 mM KCl. This medium was supplemented with 1.5 mL/L trace metal solution, as described by Vishniac and Santer [25]. The pH of the growth medium was adjusted to 8.0 by a few drops of 5M KOH.

#### 2.3.2. Growth Conditions: 5 L Enrichment Bioreactor under a Feast–Famine Regime

For the enrichment of PHA-accumulating bacteria on lab-scale, a 5 L glass autoclavable bioreactor (Applikon, Delft, The Netherlands) was operated under a feast–famine regime [9,24]. The operational set-up was as follows: The total cycle was 24 h with a feast-phase of approximately 4 h. Every 24 h (excluding the weekend), half of the culture medium was refreshed. At late famine-phase, the flock-forming PHA-producing bacteria were settled for 15 min (no stirring or aeration). Part of the upper liquid (2.5 L) was drained from the bioreactor, after which the feast-phase was initiated by adding 2.5 L of fresh medium (2× stock of the medium described above) to the bioreactor. After feeding with fresh medium, the bioreactor was stirred at 300 rpm, aerated by 0.5 L air per minute, and kept at a temperature of 30 °C. The pH and the dissolved oxygen level of the bioreactor were monitored continuously, but not adjusted. If necessary, a drop of Antifoam A (Sigma-Aldrich) was added. The acetic acid concentration was measured by the LCK 365 kit from Hach Lange for two cycles. The mixed microbial consortium from this reactor was used as inoculum for further batch experiments. 

#### 2.3.3. Growth Conditions: Polyhydroxyalkanoates (PHA) Production in 2 L Bioreactor

PHA accumulation experiments were performed in 2 L glass autoclavable bioreactors (Applikon), operating under batch mode, using 10% inoculum of the enriched culture taken after >40 cycles of selection. The reactor was operated at 30 ± 1 ℃ and stirred at 150 rpm. The growth medium was the same as for the enrichment bioreactor, with minor changes. An acetic acid concentration of 20 g/L, and a C:N ratio of 10:1 (mol/mol) were used. Antifoam A was added if necessary. Samples of 40 mL were taken daily and analyzed for total biomass production and PHA content. Cells were harvested by centrifugation at 12,000 rpm (Thermo Fisher, F15-6x 100y rotor) for 10 min and lyophilized. The PHA content within the samples was analyzed by GC, see materials and methods Section 2.8.2. 

### 2.4. Bacterial Growth Conditions—Thauera aminoaromatica MZ1(T)

*Thauera aminoaromatica* MZ1(T) was ordered from DSMZ. The lyophilized cells were revived and aseptically grown in two different media. All media were sterilized by autoclaving for 20 min at 121 °C. First, the cells were grown on the rich medium recommended by DSMZ, medium 830 supplemented with 5 mL vitamin solution (of DSMZ medium 461). The cells were grown aerobically in Erlenmeyer flasks at 30 °C, 150 rpm for 4 days, or grown under the same conditions on solid agar (1.5%) plates. Secondly, *T. aminoaromatica* MZ1T was aerobically grown on a more defined medium for *Thauera aromatica,* DSMZ medium 586, with minor changes. The pH was changed to 8.0, as carbon source 10 g/L sodium acetate was added instead of sodium benzoate, KNO_3_ was omitted, and 0.5 g/L casamino acids (Difco) were added. The C:N ratio of the medium was 24.6 (mol/mol), without taking the relatively small amounts of casamino acids and vitamins into account. Cultures on medium 586 were inoculated by 1:100 (vol/vol) of a culture grown on medium 830, and subsequently grown in 200 mL medium in 1 L non-baffled Erlenmeyer flasks that were sealed with cotton plugs for a few days to a week. Analysis of the optical density (OD) of the cultures was difficult due to flock formation. *T. aminoaromatica* MZ1T grows slowly under these conditions. The cells were harvested as described above and 0.08 g of lyophilized cell pellet (cell dry mass) was obtained from the 200 mL culture. When growing the culture for longer than a week, 10 g/L sodium acetate was added to the culture, to prevent PHA degradation.

### 2.5. Chemical Extraction of PHA

The chloroform extraction method was used to extract PHA from the lyophilized cells [26,27]. First, the cells were harvested by centrifugation at 12,000 rpm (Thermo Fisher, F15-6x 100y rotor) for 10 min and then washed with distilled water. After washing, the pellet was kept at −20 °C, and then lyophilized for 24 h. For polymer extraction, 1 g dried cells were dissolved in 20 mL chloroform in a 100-mL screw-capped glass bottle and incubated at 60 °C for 72 h. The remaining suspension was filtered over a Whatman GF/A glass microfiber filter to remove the cell debris. The remaining chloroform solution was transferred to a 250-mL flask. PHA within this solution was precipitated by adding cold absolute methanol (1:9), this mixture was incubated at 4 °C for 24 h (or −20 °C for 2h). The PHA precipitate was recovered by filtration over a Whatman GF/A glass microfiber filter and air-dried overnight.

### 2.6. Microbial Characterization by Microscopy

#### 2.6.1. Phase-Contrast and Fluorescence Microscopy—Nile Blue A Staining

The PHA-producing mixed microbial consortium was regularly analyzed by phase-contrast microscopy. To analyze whether the cells contained PHA, Nile blue A staining was performed as described by Ostle and Holt, with minor changes [28]. The cells were stained on a slide by a 1% aqueous solution of Nile blue A and incubated at 55 °C for 10–15 min. After staining, the slides were washed in three steps; (1) with distilled water, (2) with an 8% acetic acid solution for 1 min, and (3) with distilled water. The slides were dried using scratch and dust-free paper and a glass cover slide was placed. The samples were analyzed with a Euromex Oxion fluorescence microscope using a green excitation wavelength of 560 nm and an emission wavelength of 595 nm.

#### 2.6.2. Cryo-Transmission Electron Microscopy (Cryo-TEM)

Bacterial samples of 3 µL were applied on a holey-carbon coated grid (Quantifoil 3.5/1) and vitrified in ethane in a Vitrobot (FEI, The Netherlands). Samples in the frozen, hydrated grid were observed by a Tecnai T20 (FEI) electron microscope operating at 200 keV, equipped with a Gatan cryo-stage (Gatan, model 626). Images were recorded under low-dose conditions on a slow-scan CCD camera.

### 2.7. Genomic Diversity Analysis

The microbial diversity of the mixed microbial consortium was studied by 16S-rDNA analysis. Genomic DNA of 1–8 mL of the consortium was extracted using the FastDNA Spin kit for soil from MP Biomedicals. The extracted genomic DNA was send for 16S-rDNA analysis to MR DNA (Shallowater, TX, USA) and analyzed by bTEFAP PacBio Sequel sequencing technology using the primer set 27F and 1492R. Diversity profiles were obtained by plotting the obtained percentage from MR DNA in a Sunburst graph.

### 2.8. Analytical Analysis of PHA

#### 2.8.1. Determination of the Acetic Acid Concentration by High-Performance Liquid Chromatography (HPLC) or Kit

Acetic acid (or acetate depending on the pH of the culture medium) was the sole carbon source added to the culture media used. The decrease in acetic acid concentration during growth was analyzed using the LCK 365 kit for organic acids from Hach Lange or by high-performance liquid chromatography (HPLC). For HPLC analysis, 2 mL samples of the culture medium were taken every 24 h [29]. Prior to injection, cell-free supernatant was obtained by centrifugation (13,500× *g*, Eppendorf Centrifuge 5424, 10 min, 4 °C), and subsequently filtered over a 0.2 µm cellulose acetate (CA) membrane. The filtered aqueous samples were analyzed with an Agilent 1200 HPLC equipped with an Agilent 1200 pump, a refractive index detector, and a standard ultraviolet detector. Samples were separated over a Bio-Rad organic acid column (Aminex HPX-87H) which was maintained at 60 °C. An eluent of 5 mM sulfuric acid was used, with a flow rate of 0.55 mL/min. The elution of acetic acid was followed at 210 nm. Calibration curves of acetic acid were prepared for accurate quantification and were based on a minimum of five data points within the range of 1–20 g/L acetic acid with an excellent linear fit (R^2^ > 0.99).

#### 2.8.2. Gas Chromatography–Mass Spectroscopy (GC–MS)

For characterization and quantitative analysis of the produced PHA, the extracted polymer was depolymerized by methanolysis and the corresponding methyl esters of the monomers were analyzed by GC–MS. Fifty mg lyophilized cells were resuspended in 2 mL chloroform, 1.7 mL methanol, and 0.3 mL 98% sulfuric acid. This mixture was incubated at 100 °C in a water bath shaker for 4 h. After cooling down, 1 mL of water was added to the reaction mixture for phase separation [30]. Benzoic acid was added as internal standard, and commercial PHB (Sigma-Aldrich) was analyzed together with the samples as an external standard. The lower organic phase, containing the methyl esters, was analyzed on a Hewlett-Packard 6890 gas chromatograph equipped with a Rxi-5Sil capillary column (30 m × 0.25 mm i.d. and 0.25 µm film thickness) and a Quadrupole Hewlett-Packard 5973 mass selective detector. Helium was used as carrier gas at a flow rate of 2 mL/min. Samples of 5 µL were injected at an inlet temperature of 60 °C. The oven temperature was kept at 60 °C for 3 min and then increased to 280 °C at a rate of 12 °C/min and held at 280 °C for 8 min [31]. All samples were diluted 10 times with chloroform and filtered over a 0.2 µm PTFE membrane prior to analysis.

#### 2.8.3. Nuclear Magnetic Resonance (NMR)

For the characterization of the polymer, 10 mg of the extracted biopolymer was dissolved in 2 mL deuterated chloroform (CDCl_3_). As a standard, commercial PHB (Sigma) was analyzed together with the samples. ^1^H NMR spectra were recorded by an Agilent Technologies 400 MHz MR-DD2 supplemented with a 5 mm One nuclear magnetic resonance (NMR) probe, or by a Varian Mercury Plus 300 MHz supplemented with a 5 mm 4nuc probe. Settings: 45° duration of pulse, 1 s repetition delay, 8–32 scans, and an acquisition time of 2.923 s (400 MHz) or 1.953 s (300 MHz).

## 3. Results and Discussion

### 3.1. Description of the Mixed Microbial Consortium

**Figure 2:**
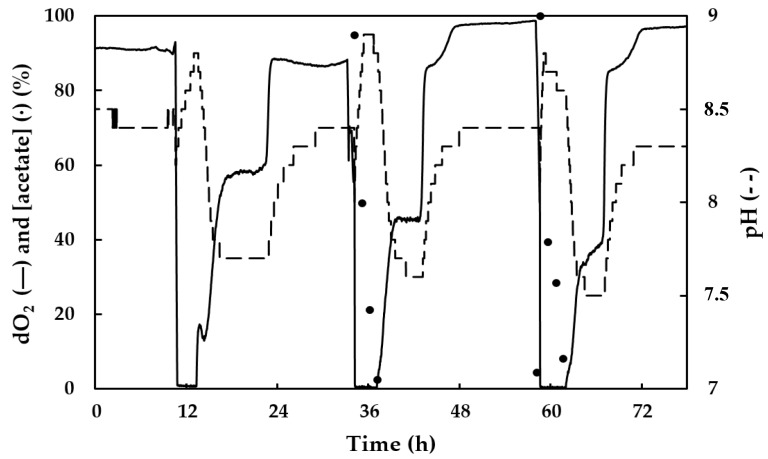
Daily pattern of the pH (dashed line) and dissolved oxygen concentration (solid line) of a polyhydroxyalkanoate (PHA)-producing mixed microbial consortium under a steady state feast–famine regime upon feeding with acetate (dots). An acetate concentration of 100% corresponds to 500 mg/L acetic acid.

To enhance the selection of the PHA-producing bacteria from the pilot reactor in our 5 L bioreactor, a feast–famine regime was combined with gravitational settling. The PHA-producing bacteria appeared to be floc forming and settling. The culture was allowed to settle daily before the medium was exchanged from the top of the bioreactor. If the amount of biomass became too much, the excess was removed from the reactor. The pH and the dissolved oxygen level were recorded continuously. Upon feeding with acetate, the dissolved oxygen level dropped to zero due to the increased metabolism of the culture. When acetate became limited, the dissolved oxygen level increased again. The oxygen limiting conditions lasted 5 h at most. The pH showed a repetitive cycle upon feeding too. The pH became first more alkaline, then more acidic, and subsequently recovered to a pH value of 8–8.5. PHB production by this mixed microbial consortium was confirmed. To further enrich the PHA-producing bacterial strain(s), the 5 L bioreactor was operated under these conditions (feast–famine regime with gravitational settling and oxygen limitation period) for several months, thereby obtaining a steady-state with a stable repetitive pattern, as shown in Figure 2.

### 3.2. Culture Growth and PHA Production by the Mixed Microbial Consortium

**Figure 3:**
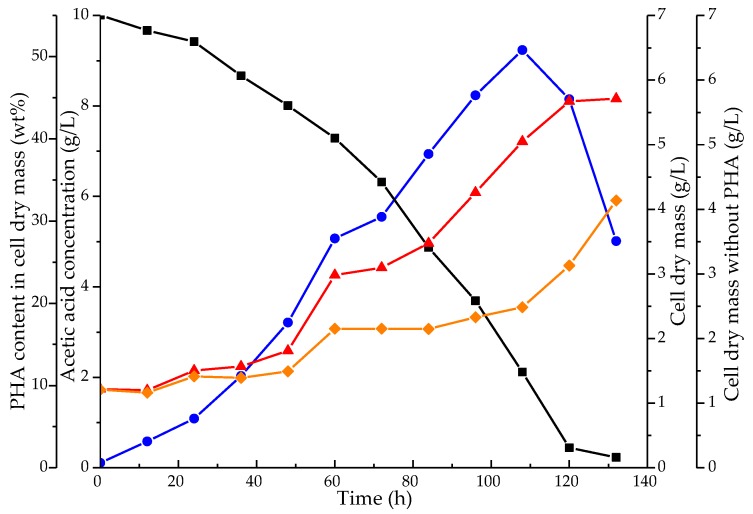
PHA accumulation by the mixed microbial consortium when grown in a 2 L batch bioreactor, with 10 g/L acetic acid as the carbon source and a C:N ratio of 10:1 (mol/mol). The acetate concentration is shown in black squares, the PHA content of the dried cells (wt%) in blue circles, and the cell dry mass including and excluding PHA in red triangles and orange diamonds, respectively.

To study the production of PHA by the mixed microbial consortium, the consortium was grown in a 2 L batch bioreactor using 10 g/L acetic acid as the sole carbon source and a C:N ratio of 10:1 (mol/mol). Samples were taken every 12 h and analyzed for the substrate concentration, total cell dry mass (CDM), and for the PHA content within the CDM, see Figure 3. The PHA content within the dried biomass increased gradually during bacterial growth, reaching a maximum amount of PHA within the cell dry mass of 50.4% after 110–120 h. When acetate depleted, a decrease in the PHA content of the dried biomass was observed. This pattern was reproducible and observed before by other researchers [5]. Besides PHA synthases for biopolymer production, these bacteria can produce PHA depolymerases to initiate its metabolization. To prevent PHA degradation, the PHA depolymerase(s) could be knocked out, as was done for *Pseudomonas putida* KT2442 and *Rhodobacter sphaeroides* HJ [32,33].

### 3.3. Extraction and Identification of PHA

In order to study the PHA produced, it was extracted from the bacterial cells. Here, we used a chloroform extraction method, as described in the materials and methods section. A white product was obtained after chloroform extraction of the lyophilized red-brown colored cell pellets, see Figure S1 in the supplementary materials. Using both GC and ^1^H NMR, the extracted product was confirmed to be pure PHB, see Figure S2 and Figure S3. The production of pure PHB was expected since the consortium was fed with acetate as the sole carbon source.

### 3.4. Genomic (Diversity) Analysis of the Mixed Microbial Consortium

**Figure 4:**
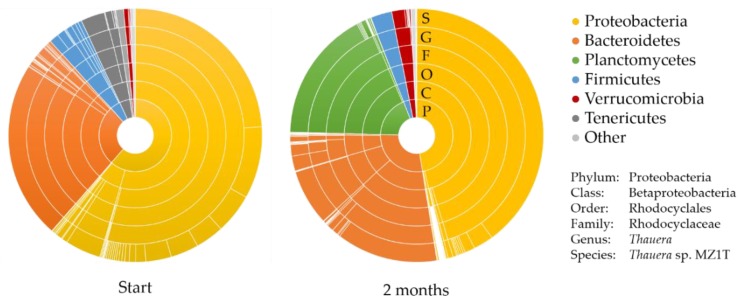
Sunburst plots of the genomic diversity of the PHA-producing mixed microbial consortium at the start (left) and after 2 months (right) of enrichment in the 5 L lab-scale selector-bioreactor.

In order to identify the predominant PHA-producing organism in the mixed microbial consortium, probably being a flock forming PHB-producing bacterium that settles by gravity, the genomic diversity of this mixed microbial consortium was studied by 16S-rDNA analysis. Two samples were analyzed, one of the original consortium retrieved from the industrial pilot reactor and one after two months of enrichment in the 5 L lab-scale selector. In the pilot reactor, several *Thauera spp*. were abundantly present, with one dominating with 24% of the total community. This predominant species became even more predominant after two months of growth in the feast–famine operated 5 L selector-reactor. An increase from 24 to approximately 40% was observed, see Figure 4 and Table 1 and Table 2. However, the abundance of the other *Thauera* species reduced significantly over the two months. The predominant species was further identified, showing >99.9% homology with *Thauera aminoaromatica* MZ1T [34,35]. Additionally, a new group of organisms increased significantly after several months of enrichment: the planctomycetes, ubiquitous bacteria that live in all sorts of habitats [36]. The dominating species in this group is *Algisphaera* spp., belonging to the class *Phycisphaerae*, known to be associated with aquatic phototrophs [37]. Since these phototrophs were not present in detectable amounts in the original PHA-producing industrial pilot reactor, we did not focus on these microorganisms for their involvement in PHA production. The relative abundance of all species belonging to the Bacteroidetes-phylum did not change significantly during the enrichment-period, either. Therefore the focus of our study was on the proteobacteria, and on the *T. aminoaromatica sp.* specifically.

*T. aminoaromatica* MZ1T is a Gram-negative beta-proteobacterium belonging to the family of the *Rhodocyclaceae*. Sayler deposited this species in the German collection of microorganisms and cell cultures (DSMZ) under no. 25461 [34]. *T. aminoaromatica* MZ1T was originally isolated from activated sludge mixed liquor from the Eastman Chemical company wastewater treatment facility (Kingsport, TN, USA) [34]. This species was shown to be a floc-forming facultative anaerobic strain capable of producing abundant amounts of exopolysaccharide (EPS) [35,38]. The ability to form flocs may be an important aspect in explaining why especially this *Thauera* species became more dominant in our selector-vessel: the selective pressure was towards the capability of storing reserve materials as PHA due to the feast–famine regime on the one hand, and on the capability of gravitational settling possibly due to floc-formation on the other hand (see above). A closely-related strain belonging to the same species, *T. aminoaromatica* S2, has been previously studied [35,39,40].

Kutralam-Muniasamy et al. analyzed the fully sequenced genomes of 66 beta-proteobacteria for the presence of a genetic pathway for PHA production [41], one of these being the genome of *T. aminoaromatica* MZ1T, that was fully sequenced by Jiang et al. [35]. They showed that *T. aminoaromatica* MZ1T contains a pathway for PHA production. When blasting the genes known to be involved in PHA production in *Cupriavidus necator* H16 to the genome of *T. aminoaromatica* MZ1T homologs of most genes were found, see Table 3. These genes cover the PHA synthesis genes (*pha*A, acetyl-CoA acetyltransferase; *pha*B, acetoacetyl-CoA reductase and *pha*C, PHA synthase), a gene for phasin expression (*pha*P, a surface protein of the PHA granules), a regulatory gene for phasin expression (*pha*R) and a PHA depolymerase (*pha*Z).

### 3.5. Does T. aminoaromatica MZ1T Produce PHA?

To study the ability of *T. aminoaromatica* MZ1T to produce PHA, the strain was obtained from DSMZ (no. 25461, called *T. aminoaromatica* MZ1, showing >99.9% resemblance with the dominant species in our selector-reactor). When grown in batch culture, it was observed that the organism initially grew as homogeneous suspension, but after several days floc formation occurred, see Supplementary materials Figure S4. This floc forming capacity was previously shown [35,38].

The production of PHB by *T. aminoaromatica* MZ1T was studied by exchanging the rich growth medium recommended by DSMZ (medium 830) for an adapted version of the defined medium recommended for *Thauera aromatica* (medium 586). This medium allowed us to grow the strain on a medium that was more comparable to the medium used for the mixed microbial consortium in the 5 L selector-bioreactor. The culture grew slowly but steadily on this medium, and after a few days, flocs appeared. The production of PHB was analyzed by cryo-TEM on the whole cells, and by ^1^H NMR on the extracted biopolymer.

**Figure 5:**
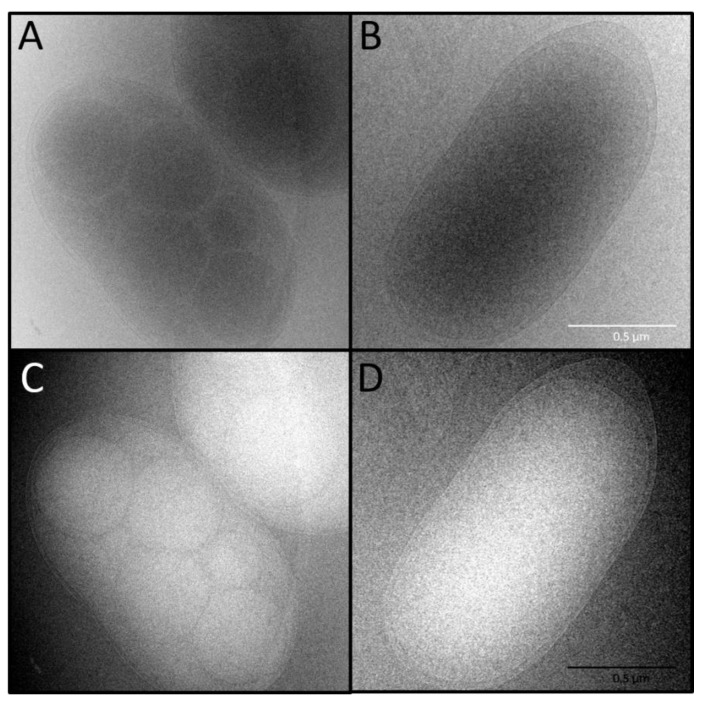
Cryo-TEM of *T. aminoaromatica* MZ1T cells with (panel (**A**)) and without polyhydroxybutyrate (PHB) (panel (**B**)). Panels (**C**) and (**D**) are negative images of panels (**A**) and (**B**) made in GIMP with a slightly adjusted contrast.

When *T. aminoaromatica* MZ1T was grown on the rich DSMZ medium 830 the cryo-TEM images showed nicely uniform rod-shaped bacterial cells with a size of approximately 1 μm × 2 μm, see Figure 5. Remarkably, when the cells were grown on the defined medium, multiple dense spheres of 0.2–0.5 µm where visible within the bacterial cells. This result suggests that *T. aminoaromatica* MZ1T stores some compounds within their cells under nitrogen limiting conditions, possibly being PHA. Applying chloroform-based extraction procedures for extraction of PHA and subsequent analysis of the extracted material by ^1^H NMR confirmed this hypothesis: *T. aminoaromatica* MZ1T produced PHB when grown on acetate under nitrogen limiting conditions, see Supplementary materials Figure S5. This is the first evidence showing that *T. aminoaromatica* MZ1T is capable of PHA production as supposed by the presence of the PHA-producing genes in the genome as shown by Kutralam-Muniasamy et al. [41].

In this research, a PHA-producing mixed microbial consortium was studied and characterized. 16S-rDNA analysis showed that the predominant species within the consortium is the beta-proteobacterium *Thauera aminoaromatica* MZ1T. This bacterial species was isolated and characterized before [34,38], but not fully studied yet. Its genome was fully sequenced, and a pathway for PHA production had been identified previously [35,41]. We demonstrated that *T. aminoaromatica* MZ1T is indeed capable of PHA production under aerobic nitrogen limiting conditions. It produces PHB when grown on acetate as the sole carbon source, comparable to other PHA-producing bacteria [20]. This strain grows well, both as monoculture and within a natural mixed microbial consortium in which it could be enriched to at least 40%. A striking characteristic of this strain is its floc-forming ability and gravitational settling, allowing to separate the bacterial flocs from the majority of the medium just by gravity. This allows a relatively easy selective production of high volumes of PHA-producing biomass.

Besides PHA, *T. aminoaromatica* MZ1T is also reported to be able to produce large amounts of other biopolymers like exopolysaccharide (EPS) [38]. It would be interesting to study if *T. aminoaromatica* MZ1T is also capable of simultaneous production of both EPS and PHB, as previously shown for other bacteria [42], and how to direct production towards the most valuable polymer.

By identifying *Thauera aminoaromatica* MZ1T as a key-microbe in an industrial mixed microbial consortia (MMC) that produces PHA from organic waste, we will be able to further characterize this organism in relation to its PHA-producing capacities. For example, to optimize culturing conditions and substrate use to increase PHA production yields, and to direct the (co)-polymer composition. Knowledge of the cell-membrane composition and the structures of the PHA-granules will help us to develop more environmentally friendly extraction methods to keep the green label of this biopolymer.

## 4. Conclusions

In this work, we identified *Thauera aminoaromatica* as the dominant microorganism in a PHA-producing MMC from an industrial pilot plant. This Gram-negative beta-proteobacterium, belonging to the order of the Rhodocyclales, was further enriched by a feast–famine regime and gravitational settling from 24% to 40% in two months. Using cryo-electron microscopy analysis, intracellular dense granules, possibly consisting of PHB, were observed when grown on acetate as the sole carbon source and a C:N ratio of the medium of 10:1. NMR analysis confirmed PHB production by *T. aminoaromatica* MZ1T. Identification and characterization of the key-microbe(s), such as *T. aminoaromatica* MZ1T within PHA-producing MMCs, allows for a better understanding of PHA production in terms of yield and polymer composition.

## Figures and Tables

**Table 1:** Top 10 bacterial species as determined by 16S-rDNA analysis of the mixed microbial consortium of the pilot plant used to start the 5 L selector-bioreactor in our lab.

	Species	Phylum	Relative Abundance (%)
1	*Thauera* sp. MZ1T *	Proteobacteria	23.9
2	Uncultured *Haliscomenobacter* sp.	Bacteroidetes	22.7
3	Uncultured *Thauera* sp.	Proteobacteria	9.2
4	*Thauera aminoaromatica*	Proteobacteria	5.1
5	*Micavibrio aeruginosavorus* arl_13	Proteobacteria	4.5
6	*Thauera aminoaromatica*	Proteobacteria	3.6
7	*Thauera phenylacetica*	Proteobacteria	3.6
8	Uncultured *Thauera*	Proteobacteria	3.3
9	Uncultured *Asteroleplasma* sp.	Tenericutes	3.1
10	Uncultured *Thauera* sp.	Proteobacteria	1.3

* The most abundant species showed >99.9% homology to *Thauera* sp. MZ1T (DSMZ strain 25461).

**Table 2:** Top 10 bacterial species as determined by 16S-rDNA analysis for the mixed microbial consortium after 2 months of growth in our 5 L lab-scale selector-bioreactor.

	Species	Phylum	Relative Abundance (%)
1	*Thauera* sp. MZ1T *	Proteobacteria	39.9
2	*Algisphaera agarilytica*	Planctomycetes	16.8
3	Uncultured *Ohtaekwangia* sp.	Bacteroidetes	13.0
4	Uncultured *Alkaliflexus* sp.	Bacteroidetes	7.1
5	Uncultured *Thermodesulfobium* sp.	Firmicutes	2.7
6	*Thauera aminoaromatica*	Proteobacteria	2.5
7	*Sediminibacterium salmoneum*	Bacteroidetes	1.9
8	*Prosthecobacter vanneervenii* str. dsm 12252	Verrucomicrobia	1.6
9	Uncultured *ferruginibacter* sp.	Bacteroidetes	1.4
10	*Thauera aminoaromatica*	Proteobacteria	1.3

* The most abundant species showed >99.9% homology to *Thauera* sp. MZ1T (DSMZ strain 25461).

**Table 3:** Gene equivalents for PHA synthesis observed in the genome of *T. aminoaromatica* MZ1T. The genes known to be involved in PHA synthesis of *Cupriavidus necator* H16 [41] were blasted to the genome of *T. aminoaromatica* MZ1T.

Category	Genes	Description and Function	NCBI/GenBank Code *C. necator* H16 [41]	NCBI/GenBank Code *T. aminoaromatica* MZ1T
Synthesis	*pha*A	Acetyl-CoA acetyltransferase	CAJ92573.1	ACK53504.1 ACK53575.1 ACK53579.1 ACR01093.1
	*pha*B	Acetoacetyl-CoA reductase	CAJ92574.1	ACK53788.1 ACR01719.1
	*pha*C	PHA synthase	CAJ92572.1 CAJ93103.1	ACK53500.1 Class I ACK53786.1 Domain protein ACK53908.1 Domain protein
Surface proteins	*pha*P	Phasin	CAJ92517.1	ACR02450.1 ACR01124.1 ACK54768.1 ACK54704.1 ACK53642.1
Gene regulation	*pha*R	PHA repressor, regulates phasin expression	CAJ92575.1	ACR01718.1
Degradation	*pha*Z	PHA depolymerase	CAJ92291.1	ACK52971.1 PHA depolymerase ACK53308.1 Esterase

## Supplementary Materials

The following are available online at https://www.mdpi.com/2306-5354/7/1/19/s1 Figure S1. PHA extracted from the mixed microbial consortium grown on acetate, Figure S2. Gas chromatogram of methanolized commercial PHB and PHB produced by the mixed microbial consortium, Figure S3. ^1^H NMR (400 MHz, CDCl_3_) spectrum of commercial PHB and PHB produced by the mixed microbial consortium, Figure S4. Flock formation of *T. aminoaromatica* MZ1T, Figure S5. ^1^H NMR of extracted PHB produced by *T. aminoaromatica* MZ1T.
