# Supplementary material for: Thauera aminoaromatica MZ1T Identified as a Polyhydroxyalkanoate-Producing Bacterium within a Mixed Microbial Consortium
Source: Bioengineering (Basel). 2020 Feb 21;7(1):19. doi: 10.3390/bioengineering7010019 (PMC7175198; doi:10.3390/bioengineering7010019)
Supplement: Supplementary file 1 [file bioengineering-07-00019-s001.pdf]

Supplementary materials

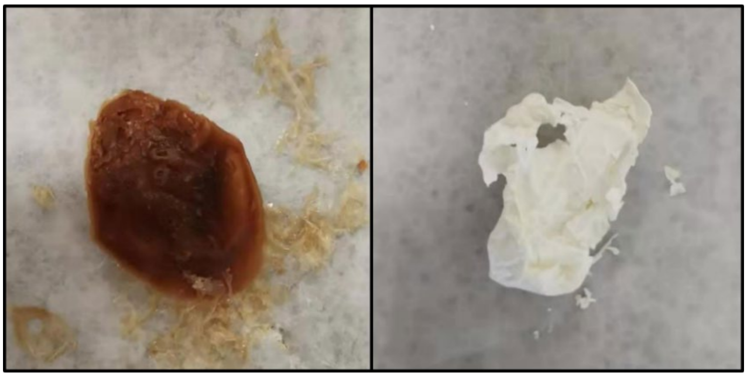

Figure S1. PHA extracted from the mixed microbial consortium grown on acetate. The lyophilized cell pellet is shown left, and the chloroform extracted PHA on the right.

The by chloroform extraction extracted white product (see Figure S1) was studied and characterized by GC and ^{1}H NMR. Since the culture was only fed with acetate, the biopolymer is expected to be poly-3-hydroxybutyrate (PHB). For GC analysis, the biopolymer was decomposed into its monomers by methanolysis. If solely PHB is produced, the methanolized product will be methyl 3-hydroxybutyrate (methyl-3HB). As comparison poly[(*R*)-3-hydroxybutyric acid] (PHB) bought from Sigma-Aldrich was analyzed as reference compound, using the same methods. The GC results clearly showed that the extracted biopolymer is pure and identical to the commercial PHB, both revealing a peak with a retention time of 4.074 min for methyl-3HB, see Figure S2. ^{1}H NMR confirmed this result and an identical profile for both the extracted biopolymer and commercial PHB was obtained, see Figure S3. From these results, it was concluded that the studied mixed microbial consortium was able to accumulate PHB when grown under nitrogen limiting conditions with acetate as the sole carbon source.

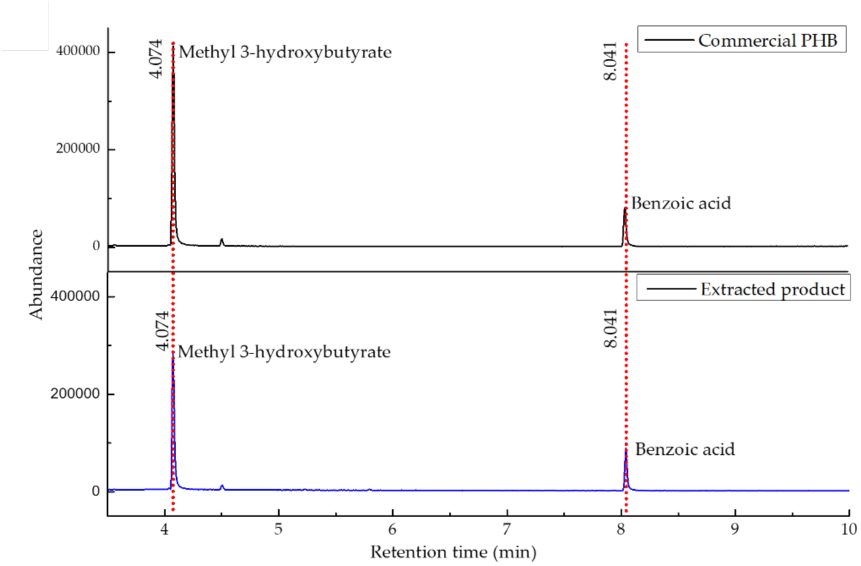

Figure S2. Gas chromatogram of methanolized commercial PHB (upper panel) and PHB produced by the mixed microbial consortium (lower panel). Both panels show the methyl ester methyl 3-hydroxybutyrate (methyl-3HB) as the degradation product of the methanolysis. Benzoic acid was added as an internal standard.

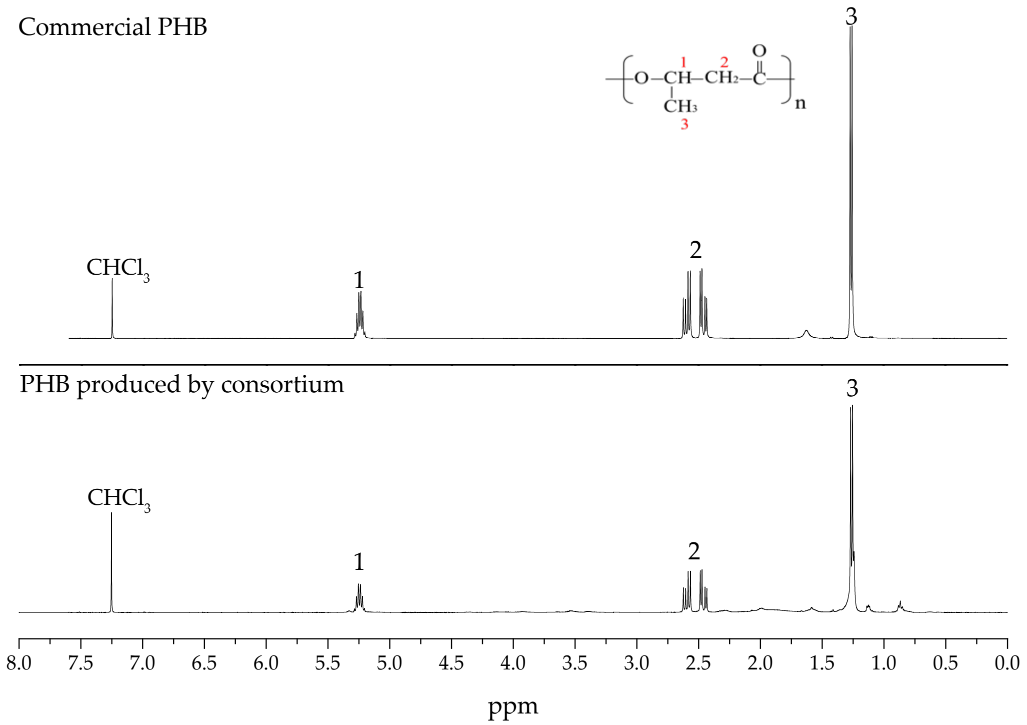

Figure S3. ^{1}H NMR (400 MHz, $CDCl_3$) spectrum of commercial PHB (upper panel) and PHB produced by the mixed microbial consortium (lower panel). The peak positions were 5.24 ppm for the -CH-group, 2.59 ppm for the $-CH_2$-group, and 1.26 ppm for the $-CH_3$ group.

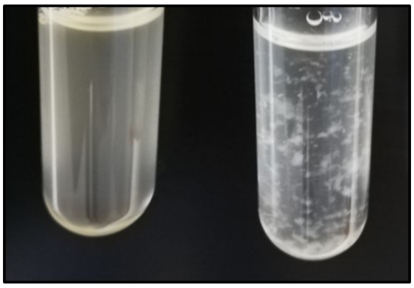

Figure S4. Flock formation of *T. aminoaromatica* MZ1T when grown on 5 mL DSMZ medium 830. The culture grows homogeneous at first (left tube). After several days, floc formation started (right tube).

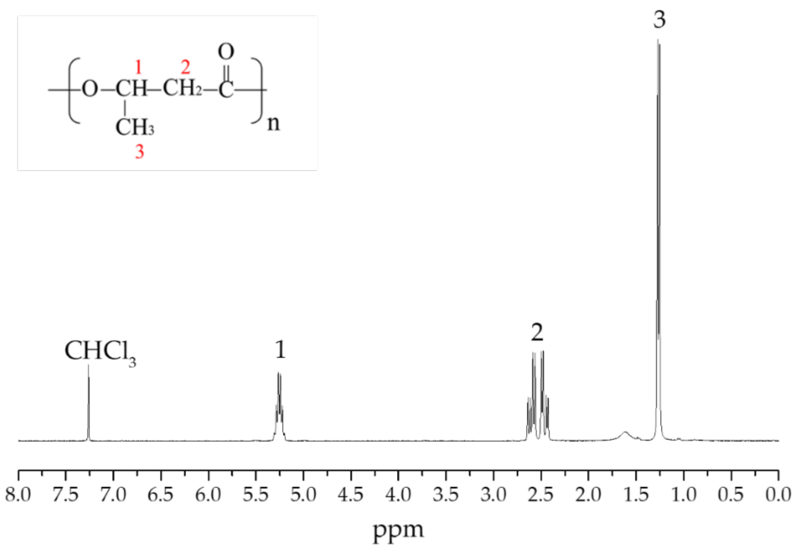

Figure S5. ^{1}H NMR of extracted PHB produced by *T. aminoaromatica* MZ1T. 1H NMR: (300 MHZ, $CDCl_3$) δ 5.25 (h, *J* = 6.3 Hz, 1H), 2.74-2.36 (m, 2H), 1.27 (dd, *J* = 6.3, 1.8 Hz, 3H). See Figure S3 for comparison with commercial PHB.
